# Screening of Plants Used as Traditional Anticancer Remedies in Mkuranga and Same Districts, Tanzania, Using Brine Shrimp Toxicity Bioassay

**DOI:** 10.1155/2018/3034612

**Published:** 2018-11-14

**Authors:** Daniel Z. Matata, Olipa D. Ngassapa, Francis Machumi, Mainen J. Moshi

**Affiliations:** ^1^Department of Natural Products Development and Formulations, Institute of Traditional Medicine, Muhimbili University of Health and Allied Sciences, P.O. Box 65001, Dar es Salaam, Tanzania; ^2^Department of Pharmacognosy, School of Pharmacy, Muhimbili University of Health and Allied Sciences, P.O. Box 65013, Dar es Salaam, Tanzania; ^3^Department of Biological and Preclinical Studies, Institute of Traditional Medicine, Muhimbili University of Health and Allied Sciences, P.O. Box 65001, Dar es Salaam, Tanzania

## Abstract

**Background:**

Inadequate specialized cancer hospitals and high costs are contributing factors that delay cancer patients from accessing health care services in Tanzania. Consequently, majority of patients are first seen by Traditional Health Practitioners (THPs) before they access specialized services. This study presents ethnomedical information and preliminary evaluation of 25 plant species claimed by THPs in Mkuranga and Same districts of Tanzania on use for treatment of cancer. Literature search and laboratory investigation results are presented to support evaluation.

**Methods. This study was a single disease ethnomedical enquiry focusing on plants being used for cancer treatment:**

Face-to-face interviews and questionnaires were administered to* eight (8) THPs* in Mkuranga and Same districts on the claimed plants and their use for management of cancer. Plants were selected based on being frequently mentioned and emphasis given by THPs. Literature search and brine shrimp toxicity (BST) of methanol : dichloromethane (1:1) extracts was used as surrogates to evaluate strength of the claims.

**Results:**

This study reports 25 plant species used by the THPs in two districts of Tanzania. Eight plants (32%) have been reported in the literature to have activity against cancer cells. BST results revealed, 14 (56%) plants exhibited high toxicity against brine shrimps. The most active plants included* Croton pseudopulchellus *Pax (LC_50_ 4.2 *μ*g/ml),* Dalbergia melanoxylon *Guill. & Perr. (LC_50_ 6.8 *μ*g/ml),* Loranthus micranthus* Linn (LC_50_ 4.0 *μ*g/ml),* Ochna mossambicensis *Klotzsch (LC_50_ 3.3 *μ*g/ml), and* Spirostachys africana* Sond. (LC_50_ 4.4 *μ*g/ml); their toxicity was comparable to that of* Catharanthus roseus *(L) G. Don. (LC_50_ 6.7 *μ*g/ml), an established source of anticancer compounds. Nine other plants had LC_50_ values between (19.8 and 71.6) *μ*g/ml, indicating also potential to yield anticancer.

**Conclusion:**

Literature search and BST results provide a strong support of the potential of the claimed plants to yield active anticancer compounds.

## 1. Introduction

Cancer is a major public health problem affecting both developing and developed nations, but whereas 50% of cancer patients in developed countries die of the disease, in developing countries 80% of cancer victims are diagnosed with late-stage incurable tumors [1]. In the same vain while the incidence of cancer in Africa is about one-third that of North America, the death rate is nearly the same, thus further illustrating the cancer treatment challenges that African countries face and the need to develop solutions for addressing this challenge.

According to the Foundation for Cancer Care, an estimated 30,000 new cancer cases are diagnosed each year, in Tanzania, but there is only one specialized cancer treatment center to serve the entire population of nearly 55 million people, with a capacity to see only 5000 patients in a year. Hence, majority of patients access treatment too late, when their cancers have already advanced and are at stages for which the only remaining option is palliative care [2, 3]. Lack of cancer education, inadequate screening services, and detection methods contribute to late diagnosis and access to treatment [3, 4]. The Ocean Road Cancer Institute, situated on the eastern coast of the country, in Dar es Salaam, is the only specialized cancer treatment hospital for the whole country; hence patients have to incur high transport costs to reach the center, and this, combined with the high costs of anticancer drugs, leaves the majority of patients with no option, but to depend on Traditional Health Practitioners (THPs) to care for them.

Anecdotes abound of successful treatment of cancers by THPs in Tanzania, but only a few of the plants that are used have been evaluated for efficacy using cancer cell lines [5–7] and some have only been evaluated using proxy assays such as the brine shrimp lethality test [8–12]. Two studies on Tanzanian plants are particularly inspiring to efforts to screen Tanzanian plants for anticancer activity [13, 14]. In one study, aguiaine sesquiterpene, Englerin A, was isolated from a Tanzanian medicinal plant,* Phyllanthus engleri *Pax (Euphorbiaceae), which has been demonstrated to have selective activity against kidney cancer cells [13]. More recently isoflavone derivatives, rhynchoviscin, genistein, sophoraisoflavone, licoisoflavone A, and 3'O-methylorobol with antiangiogenic activity and hence potential for cancer treatment, were isolated from another Tanzanian plant,* Rhynchosia viscosa* (Roth) DC that is used in traditional medicine [14].

We report in this study documentation of 25 plant species that are used by THPs in Mkuranga district (Coast Region) and Same district (Kilimanjaro Region) of Tanzania, for treatment of cancer. We also used information from the literature and results of their toxicity profile against brine shrimp larvae (*Artemia salina*) as surrogates for interrogating their potential as sources of anticancer compounds.

## 2. Materials and Methods

### 2.1. The Study Area

The study was done in Mkuranga district (Coast region) and Same district (Kilimanjaro region) located on the eastern coast and north-eastern parts of Tanzania, respectively. Selection of the two areas was based on information from patients who visited Traditional Health Practitioners in the two districts to seek treatment for cancer as well as** other informants and local government leaders**. There is no documented evidence of successfully treated patients but there are famous THPs who are frequently being consulted by cancer patients for their remedies in these two areas.

### 2.2. Documentation of Ethnomedical Information and Selection of Plants for Testing

Physical visits were made to the two areas and meetings convened with Traditional Health Practitioners who were interviewed orally and, then, requested to fill questionnaires concerning their knowledge on treatment of cancer. Information documented included age, how they became experts in cancer treatment, how they identify cancer patients, the medicinal plants used, and methods of preparation. Traditional healers who seemed knowledgeable and claimed to treat cancer patients were selected for further discussion.

### 2.3. Selection and Collection of Plants for Biological Testing

Selection of plants for documentation was based on the number of practitioners who used the same plants for treatment of cancer. Plant materials were collected, labelled, and kept away from direct sunlight to minimize metabolic degradation and the effects of ultra violet light. In all cases, a botanist from the Botany Department, University of Dar es Salaam, assisted in the identification and preparation of voucher specimens for plants of interest. Collected plant materials were dried in open air at room temperature and away from direct sunlight.

### 2.4. Information from the Literature

Literature search was done to determine if any of the plants was being used somewhere else for treatment of cancer or had been screened for anticancer activity. The information obtained is reported as information that supports the Traditional Health Practitioners' claims, but where there is no information the interpretation was taken as an indication for need of studies to establish proof having anticancer activity.

### 2.5. Brine Shrimp Toxicity Testing Materials

Brine shrimp eggs were obtained from Aquaculture Innovations (Grahams Town, South Africa). Solvents and other chemical reagents used in this study (with their sources) included Dimethyl sulphoxide (DMSO; Sigma: Poole, Dorset, UK); methanol, ethanol, dichloromethane, petroleum ether, and ethyl acetate (CARLO ERBA, Van de Reut, France); sulphuric acid, sodium bicarbonate, sodium hydroxide, hydrogen peroxide, and vanillin (Fisher Scientific Ltd., Leicestershire, UK). Sea salt was prepared by evaporation of water collected from the Indian Ocean along the Dar es Salaam coast.

### 2.6. Extraction of Plant Materials

Dried plant materials were ground using a milling machine and then 100g of each powdered plant material was extracted by maceration using methanol: dichloromethane (1:1), for about 24 hours, after which the extract was decanted and filtered. The extracts were concentrated using a rotary evaporator (Heldolph instruments Gmbh, Walpersdorfer, Germany) at 40°C, under reduced pressure, after which they were dried further with a freeze-drier (Edwards, BOC Ltd., Crawley Sussex, England), to remove any traces of water. The dried crude extracts were kept in vials in a freezer at -20°C until when needed for biological tests.

### 2.7. Evaluation for Brine Shrimp Toxicity

Stock solutions (40 mg/ml) of extracts were prepared in DMSO, diluted into varying concentrations. A solution of DMSO (0.6%) in artificial sea water was used as a negative control, while a methanol: dichloromethane (1:1) leaf extract of* Catharanthus roseus* (Apocynaceae), a plant with known anticancer activity, was used as a positive control. Ten brine shrimp larvae were introduced into each vial containing 5mls of the test solution or control; after 24 hours the nauplii were examined against a lighted background and the number of live larvae counted. The mean percentage mortality was plotted against the logarithm of concentrations using the Fig P computer program (Biosoft Inc., USA), which also gives regression equations. The regression equations were used to calculate LC_16_, LC_50_, LC_84_, and 95% confidence intervals (95%CI).

## 3. Results

### 3.1. Ethnomedical Information and Proof of Concept from the Literature

Table 1 presents a list of 25 plant species that are used by Traditional Health Practitioners (THPs), in Mkuranga and Same districts, for the treatment of cancer. Among the listed plant species 22 are used in Same district, two are used in Mkuranga, and one is used in both districts. The plant species belong to 17 plant families, with 1-4 plant species, being from each family. The family Fabaceae was represented by more plant species (4) than the other families, followed by Bignoniaceae, Ebenaceae, and Malvaceae, each of which was represented by 2 plant species and one species for each of the remaining families. Information from the literature [10, 15–48] indicates that 8 of the listed plants including,* Carissa spinarum *L. [15, 16],* Markhamia obtusifolia *(Baker) Sprague [17, 18],* Kigelia africana *(Lam) Benth. [19–23],* Diospyros zombensis *(B.L Burtt) F. White [30],* Euclea natalensis *A.DC. [31],* Acacia nilotica *(L) Delile [34, 35],* Cassia abbreviata *Oliv. [5, 37], and* Ochna mossambicensis *[44, 45] have confirmed activity against one or more cancer cell lines.

### 3.2. Brine Shrimp Lethality Test

Different levels of toxicity to brine shrimps were observed in extracts of the studied 25 plant species, as shown in Table 2. Fourteen (56%) out of 25 plant species exhibited toxicity to brine shrimp larvae, with LC_50_ values of less than 100 *μ*g/ml. The results further indicate that the most active extracts (LC_50_< 10 *μ*g/ml) were those from the stem barks of* Croton pseudopulchellus *(LC_50_ 4.2 *μ*g/ml),* Dalbergia melanoxylon *(LC_50_ 6.8 *μ*g/ml), and* Spirostachys africana* (LC_50_ 4.4 *μ*g/ml), leaf of* Loranthus micranthus *(LC_50_ 4.0 *μ* g/ml), and root bark of* Ochna mossambicensis *(LC_50_ 3.3 *μ*g/ml). Their toxicity was comparable to that of* Catharanthus roseus* (LC_50_ 6.7 *μ*g/ml), which is an established source of anticancer compounds [49]. Other extracts that were toxic to brine shrimp larvae, with LC_50_ values below 100 *μ*g/ml, included* Boswellia neglecta *root bark extract (LC_50_ 27.8 *μ*g/ml),* Cordia africana *root bark extract (LC_50_ 19.8 *μ*g/ml),* Diospyros zombensis *stem bark extract (LC_50_ 67.2 *μ*g/ml),* Maerua triphylla *root bark extract (LC_50_ 57.5 *μ*g/ml),* Securidaca longipedunculata *(LC_50_ 55. *μ*g/ml),* Zanthoxylum chalybeum *root bark (LC_50_ 38.5 *μ*g/ml) and stem bark (LC_50_ 26.3 *μ*g/ml) extracts,* Baphia kirkii *root bark extract (LC_50_ 71.6 *μ*g/ml),* Euclea natalensis *root bark extract (LC_50_ 66.2 *μ*g/ml), and* Leucas martinicensis *root bark extract (LC_50_ 54.0 *μ*g/ml). The remaining plant extracts exhibited LC_50_ values that were higher than 100 *μ*g/ml.

## 4. Discussion

The purpose of this study was to seek for proof of concept supporting claims by Traditional Health Practitioners (THPs) in Mkuranga and Same districts of Coast and Kilimanjaro regions of Tanzania, respectively, for the treatment of cancer. Medicinal plants such as* Cordia africana, Croton pseudopulchellus, Mystroxylon aethiopicum, Spirostachys africana, Trichodesma zeylanicum*, and* Zanthoxylum chalybeum* were mentioned to be used for a given type of cancer by more than one practitioner, despite their different locations. Information from the literature and the brine shrimp lethality test were used as the basis for this enquiry. Information from the literature shows that eight out of the 25 plants that are used by Traditional Health Practitioners (THPs) in Mkuranga and Same districts, for treatment of cancer are substantiated to have activity against various cancer cell lines [15–23, 27–32, 35, 38, 43, 45]. These include cytotoxic or antiproliferative activity against the leukaemia HL-60 cell line [15], breast and lung cancer cell lines [16], and hepatocellular carcinoma [38]. The other substantive evidence that corroborates the THPs claims is the isolation of compounds with cytotoxic activity, such as (-)-carinol, (-)-carissanol, and (-)-nortrachelogenin from* Carissa spinarum *[16], 7-methyljuglone from* Diospyros zombensis *[30] and* Euclea natalensis *[31], and two cytotoxic bioflavonoids from* Ochna mossambicensis *[45]. Cytotoxic compounds have also been isolated from* Kigelia africana *of which furanonaphthoquinones were the most active against human breast cancer cell lines [19]. Some of the plants have exhibited antioxidant activity such as* Blighia unijugata *[48],* Securidaca longipedunculata *[46], and* Loranthus micranthus* [40].* Loranthus micranthus* contains polyphenols which exhibited antioxidant [40] and immunomodulatory activities which may play a role in the treatment of cancer [41]. Reports from the literature also suggest that some of the plants being used are potentially toxic, such as* Senecio deltoideus*, belonging to the genus* Senecio *that is known to contain the hepatotoxic pyrrolizidine alkaloids [8]. In another scenario, while the claims on* Mystroxylon aethiopicum *are supported by a confirmation that used alone, the plant exhibited low cytotoxic activity against three cancer cell lines [7]; the Traditional Health Practitioners mix it with* Senecio deltoideus, *thus making this combination potentially toxic due to the presence of pyrrolizidine alkaloids [27]. Another school of thought may purport that such mixing is intended to enhance activity and minimize toxicity, but certainly this remains a subject for further studies.

In our previous studies we have shown that brine shrimp toxicity results frequently predict presence of cytotoxic activity against cancer cell lines [9, 10, 12]. Thus, from the brine shrimp results* Catharanthus roseus, *which is a known plant with anticancer activity [49], gave a LC_50_ of 6.7 *μ*g/ml in support of these previous observations [9, 10, 12]. Therefore the brine shrimp results of other plants which are likely to have anticancer activity are those with LC_50_ values below 100 *μ* g/ml. These include extracts of* Baphia kirkii *(LC_50_ 71.6 *μ*g/ml),* Boswellia neglecta *(LC_50_ 27.8 *μ*g/ml),* Cordia africana *(LC_50_ 19.8 *μ*g/ml),* Dalbergia melanoxylon *(LC_50_ 6.8 *μ* g/ml),* Diospyros zombensis *(LC_50_ 67.2 *μ*g/ml),* Euclea natalensis *(LC_50_ 66.2 *μ*g/ml),* Leucas martinicensis *(LC50 54.0 *μ*g/ml),* Loranthus micranthus *(LC_50_ 4.0 *μ*g/ml),* Maerua triphylla *(LC_50_ 57.5 *μ*g/ml),* Ochna mossambicensis *(LC_50_ 3.3 *μ*g/ml),* Securidaca longipedunculata *(LC_50_ 55.3 *μ*g/ml),* Spirostachys africana *(LC_50_ 4.4 *μ*g/ml), and* Zanthoxylum chalybeum *root barks (LC_50_ 38.5 *μ*g/ml) and stem barks (LC_50_ 26.3 *μ*g/ml). This is well supported by literature reports for* Diospyros zombensis* [33],* Euclea natalensis* [31], and* Ochna mossambicensis *[44]. For the other plants with LC_50_ values above 100 *μ*g/ml, if at all they have anticancer activity, this may probably be ascribed to other mechanisms such as arrest of the cell cycle and induction of apoptosis as reported for* Acacia nilotica* [34] or inhibition of angiogenesis. In the absence of more evidence the other plants remain to be subject for further research to determine their efficacy.

## 5. Conclusion

Reports from the literature and brine shrimp lethality test have provided preliminary information for evaluation of claims from some Traditional Health Practitioners of Mkuranga and Same districts in Tanzania, for use of some medicinal plants in the management and treatment of cancer. These results make a strong case for continuing research on most of the plants that are used by THPs in these two districts to explore possibility of isolating novel anticancer compounds.

## Figures and Tables

**Table 1:** Plants used by Traditional Health Practitioners of Mkuranga and Same districts.

**Family/Species** **[Voucher No.]**	**Vernacular name (Tribe)**	**Life form**	**Type of cancer treated (No. of THPs)**	**Part(s) Used**	**Methods of preparation and administration**	**Related ethnomedical claims or pharmacological activity reported**
**Apocynaceae**/*Carissa spinarum *L. [ DZM 16]	Mshohwe (Pare)	S	Leukemia (1) Colon cancer (1), Breast cancer (1) Cervical cancer (1)	R	Root barks are mixed with dry stem bark of *Spirostachys africana*, Sond boiled and a tea cup of the concoction is drunk three times daily.	*Carissa spinarum *stem aqueous extract (CSE) and its n-butanol fraction (CSF) inhibited cell proliferation of various human cancer cell lines in which leukaemia HL-60 cells treated with CSF showed maximum growth inhibition with IC_50_ value of 34.58±0.91 *μ* g/ml [15]. In another study the lignans (-)-carinol, (-)- carissanol and (-) nortrachelogenin isolated from the stem exhibited cytotoxic activity against breast (MCF7) and lung (A549) cancer cells [16].

**Bignoniaceae/** *Markhamia obtusifolia *(Baker) Sprague [DZM31]	Mtalanda (Pare)	T	Breast cancer (1) Cervical cancer (1)	SB	Boiled stem bark concoction is drunk. A tea cup is taken daily.	The aqueous extract of the leaves exhibited low toxicity on cell lines [17]. Low cytotoxicity was exhibited by an ethanol extract of the leaves of *Markhamia tomentosa *on HeLa and MCF-7 cancer cell lines, but the extract induced arrest of cell cycle at G1/G0 phase in HeLa, causing apoptosis within 24 h of exposure [18].

**Bignoniaceae/** *Kigelia africana *(Lam) Benth. [DZM 41]	Mmomwe (Pare)	T	Breast cancer (1) Liver cancer (1) Colon cancer (1)	SB	Stem bark mixed with approximately equal weights root barks of *Maclura africana* (Bureau)corner* Harrisonia abyssinica *Oliv and *Warburgia stuhlmannii *Engl., boiled together and one tea cup of the concoction is drunk twice a day.	The plant has yielded compounds with anticancer activity [19–21]. The fruit powder was used in the management of Polycystic Ovary Syndrome (PCOS) in two patients. [22]. An 80% methanol extract of the roots exhibited cytotoxicity to brine shrimps with LC_50_ of 7.2 *μ*g/ml [23]

**Boraginaceae/** *Cordia africana *Lam. [DZM 20]	Ithibola vaghosi (Pare)	T	Cervical cancer (2), Colon cancer (1) Breast Cancer (1) Leukemia cancer (1)	SB	Dry outer SB grinded and powder mixed in concoction of *Hydnora. Abyssinica *A.Br. and *Loranthus micranthus *Linn. Cup of concoction is drunk for several weeks	No reported anticancer activity

**Boraginaceae/** *Trichodesma zeylanicum *(Burm.f.) R.Br. [DZM 14]	Mngurere (Pare)	S	Cervical Cancer (1) Colon cancer (2) Oesophageal cancer (1) Leukemia (1)	R, L	Decoction from boiled root bark is drunk; a tea cup is taken once daily.	*Trichodesma zeylanicum *was reported to be non-cytotoxic to cancer cell lines [24] but in another study it was shown to be a potential source of antioxidants [25].

**Burseraceae/** *Boswellia neglecta *S. Moore [DZM 32]	Mtundutwa (Pare)	**S**	Leukemia (1) Colon cancer (1)	R	A tea cup of decoction from boiled root bark is drunk twice daily.	Plants of the genus *Boswellia *are reported to have biological activities that include anticancer activity [26]. The essential oil from the gum resin of *Boswellia serrata *which is used traditionally for aromatic therapy, has also been shown to have tumour cell-specific anti-proliferative and pro-apoptotic activities [27]

**Capparaceae**/*Maerua triphylla* A.Rich. [DZM 1]	Mdudu (Pare)	S	Breast Cancer (1) Leukemia (1)	R	A tea cup of a concoction from boiled root barks is drunk twice daily.	No reported uses for cancer treatment

**Celastraceae/** *Mystroxylon aethiopicum* (Thunb.) Loes [DZM.57]	Mnyavura (Pare)	T	Cervical Cancer (2) Colon cancer (1) Oesophageal cancer (1)	SB	A stem bark is boiled with the root bark of *Senecio deltoideus *less and the concoction is taken, one bowl twice a day.	Reported to have very low cytotoxic activity against three cell lines [7].

**Compositae/** *Senecio deltoideus *Less. [DZM 58]	Mkonte ( Pare)	CL	Cervical cancer (1) Colon cancer (1) Oesophageal cancer (1)	L	Leaves wrapped in fresh banana leaf and warmed on fire then squeezed to obtain a fluid which is given to kids; one teaspoonful, three times daily.	Reported as one of plants used by the Xhosa of South Africa for treatment of oesophageal cancer [28]. However, it is worth noting that plants of this genus are reported to have pyrrolizidine alkaloids which are very toxic [29].

**Ebenaceae**/*Diospyros zombensis *(B.L. Burtt) F.White [DZM 35]	Mdaa (Pare)	T	Breast Cancer (1) Leukemia (1)	SB	Concoction from boiled stem bark is drunk; one tea cup three times daily.	Two naphthoquinones, 7- Methyljuglone and isodiospyrin, extracted from *Diospyros zombensis *showed cytotoxic activities against human colon carcinoma cells.7- Methyljuglone had a LD_50_ of 7.0 x10^−2^*μ*g/ml and isodiospyrin a LD_50_ of 3.8 x 10^−2^*μ*g/ml [30]

**Ebenaceae**/*Euclea* *natalensis *A.DC. [DZM 56]	Mdagha (Pare)	S	Leukemia (1)	L, R	Root barks are boiled with the root barks of *Harrisonia abyssinica *and the concoction drunk; one tea cup twice a day.	It used in South Africa for treatment of cancer and the anticancer compound, 7-methyljuglone was isolated from the root extract of *Euclea natalensis *[31].

** Euphorbiaceae**/*Croton* *pseudopulchellus* Pax [DZM 51]	Mkuang'o mbe (Pare)	S	Colon cancer (3) Cervical cancer (2) Oesophageal cancer (1) Breast cancer (1) Liver cancer (1) Breast cancer (1)	SB	A teaspoonful of fine powdered stem bark is added to a tea cup of warm water/tea/porridge and drunk twice daily.	An ethanol extract of the root bark exhibited cytotoxic activity against brine shrimps [32].

**Euphorbiaceae**/*Spirostachys africana *Sond. [DZM 28]	Mtanga (Pare), Msalaka (Swahili)	T	Cervical Cancer (2) Colon cancer (2) Oesophageal cancer (1) Breast cancer (2) Liver cancer (1)	SB, R	Paste of powder from dry stem is applied externally on a swelling. Tea cup of concoction from boiled root barks is drunk twice daily.	Contains stable bioactive compounds with high antioxidant activity [33] and an 80% methanol extract of leaves and roots exhibited cytotoxic activity against brine shrimps with a LC_50_ of 16.4 and 45.2 *μ* g/ml, respectively [23].

**Fabaceae** */Acacia nilotica *(L) Delile [DZM62]/	Mkame (Pare)	T	Cervical cancer (1)	R	Concoction from boiled root barks drunk. One bowl twice a day.	Leaf extracts exert potential anticancer activity through the growth inhibition, cell cycle arrest and the apoptosis [34]. Four lignin fractions from the stem wood exhibited high cytotoxic activity IC_50_: 2-15 *μ*g/ml against breast cancer MCF-7 cell line [35]

**Fabaceae** */Baphia kirkii* Baker [DZM 9]	Mkuruti ( Swahili)	T	Breast cancer (1) Cervical cancer (1)	SB	Stem barks boiled with root barks of *Carissa spinarum *and *Dalbergia melanoxylon *and concoction taken for a week or more	Isolated compounds Baphikixanthones A, B, C and Baphikinone. Reported need of further investigation on their medicinal values [36]

**Fabaceae** */Dalbergia melanoxylon *Guill. & Perr. [DZM 53]	Mwingo (Pare)	T	Breast cancer (1)	SB, R	Paste of grounded dry stem is applied externally on a swelling.	Fresh leaves of *Dalbergia* *melanoxylon *are used topically by the Nyamwezi of Tabora, Tanzania, for treatment of cancer [10]. An ethanol extract of the leaves exhibited cytotoxic activity to brine shrimps with an IC_50_ of 8.64 *μ* g/ml [36].

**Fabaceae**/*Cassia abbreviata *Oliv. [FMM 3631]	Mzangaze (Pare)	S	Cervical (1) Leukemia (1) Colon cancer (1)	SB	Stem bark boiled with rhizomes of *Hydnora abyssinica*. The concoction is drunk, one tea cup twice a day or the dried powder of the mixture a is added to tea/porridge	Methanol extracts from the stem bark exhibited antioxidant activity [37]. Kamuhabwa et al., showed that extracts of this plant were cytotoxic, and thus were a potential source of promising anticancer agents [5], while a recent study reported that an aqueous extract of the root bark exhibited cytotoxic activity against hepatocellular carcinoma (HCC) cells with an IC_50_ of 1.49 *μ*g/ml [38].

**Lamiaceae** */Leucas martinicensis *(Jacq)R.Br. [DZM 10]	Mrumba- vwasi (Pare)	S	Treatment of tumours	LF	Leaves are boiled and a tea cup of the concoction is drunk twice daily for a week	The leaves are used in Brazil for treatment of benign tumors [39].

**Loranthaceae** */Loranthus micranthus *Linn [DZM 40]	Kimpa- Muoža (Pare)	P	Breast Cancer (1) Leukemia (1)	L	A tea cup of a concoction from boiled leaves is drunk three times daily.	The plant contains polyphenols with antioxidant [40] and immunomodulatory properties [41] that may have a role in cancer treatment.

**Malvaceae**/*Grewia tembensis*. Fresen [DZM 39]	Mkokoro (Pare)	S	Breast Cancer (1) Leukemia (1)	Whole	The whole plant is boiled with rhizomes of *Hydnora abyssinica*. A tea cup of the concoction is drunk twice daily for two weeks.	No reported uses for cancer treatment

**Malvaceae**/*Hibiscus micranthus *L.f. [DZM 50]	Msindati (Pare)	H	Leukemia (1)	L, R	A tea cup of the decoction from boiled root barks is drunk twice daily.	In certain parts of Gujarat the fruits and flowers of *Hibiscus micranthus* are used as antitumor [42]. An ethanolic extract of aerial parts exhibited significant antitumor activity against Sarcoma 180 A cells [43].

**Ochnaceae**/*Ochna mossambicensis *Klotzsch [DZM.29]	Nkatakwa (Pare), Mkumbi (Swahili)	T	Cervical cancer (1) Breast cancer (1)	SB, R	The yellowish part from the stem bark is grinded and applied on wounds. A concoction from boiled root barks is drunk twice daily.	Crude extracts of *Ochna *species exhibited cytotoxic activity [44] and two biflavonoids with cytotoxic activity on MCF-7 breast cancer cells were isolated from the stem bark ethanol extract of another species; *Ochna macrocalyx *[45].

**Polygalaceae**/*Securidaca longipedunculata *Fresen [DZM.59]	Mbazo (P(1)are)	T	Breast cancer (1) Liver cancer (1) Colon cancer (1) Cervical (1)	SB	The stem bark is soaked in water overnight and the resulting extract is drunk, one tea cup twice daily.	Extracts have antioxidant properties [46].

**Rutaceae**/*Zanthoxylum chalybeum *Engl. [DZM.61]	Msele (Pare)	S/T	Cervical (2) Breast cancer (1)	SB, R	A concoction from boiled stem barks is drunk, one tea cup twice a day.	*Zanthoxylum chalybeum *and *L. cornuta *were found to be highly toxic with LC_50_ <500 *μ*g/ml [47].

**Sapindaceae** */Blighia unijugata* Baker [DZM 49]	Mkurutang ulue (Pare)	T	Breast cancer (1) Cervical cancer (1)	SB,L, Seeds	A mixture of stem bark, leaves and seeds, is boiled and the concoction is drunk; one bowl is taken twice a day.	Ethanolic extracts of leaves demonstrated a consistently high antioxidant activity [48].

**Life form: **T = tree; S = shrub; H = herb; P = parasitic plant; S/T = shrub/tree; CL = climber.

**Part used: **SB = stem bark; L = leaves; R = roots; whole = whole plant.

All plants involved in the study were named according to the references: http:www.theplantlist.org/ and or http://www.tropicos.org/.

**Table 2:** Brine shrimp toxicity of medicinal plants used by THPs.

**S/N**	**Plant (Part tested)**	**Part used**	**LC50 ** *μ*g/ml	**95**%** Confidence** **Intervals**
1	*Acacia nilotica*	Stem bark	432.4	246.6 – 613.5
2	*Baphia kirkii*	Root bark	71.6	54.4 – 94.2
3	*Blighia unijugata*	Leaf	539.6	349.4 – 833.2
		Stem bark	389.8	235.57 – 645.0
4	*Boswellia neglecta*	Root bark	27.8	23.7 – 32.5
5	*Carissa spinarum*	Stem bark	440.5	301.8 – 643.0
		Root bark	342.5	192.2 – 610.2
6	*Cassia abbreviata*	Root bark	140.9	108.2 – 183.4
		Stem bark	140.9	108.2 – 183.4
7	*Cordia africana*	Stem bark	185.3	133.4 – 257.6
		Root bark	19.8	13.8 – 28.2
8	*Croton pseudopulchellus*	stem bark	4.2	3.0 – 5.8
9	*Dalbergia melanoxylon*	Stem bark	6.8	4.8 – 9.8
10	*Diospyros zombensis*	Stem bark	67.2	44.7 – 101.2
		Root bark	408.6	221.5 – 753.7
11	*Euclea natalensis*	Root bark	66.2	48.1 – 91.1
12	*Grewia tembensis*	Root bark	9710.0	6406.0 – 14716.0
13	*Hibiscus micranthus*	Root bark	173.0	139.2 – 215.0
14	*Kigelia africana*	stem bark	161.4	108.8 – 239.5
15	*Leucas martinicensis*	Root bark	54.0	43.4 – 67.1
16	*Loranthus micranthus*	Leaf	4.0	3.2 – 5.1
17	*Maerua triphylla*	Root bark	57.5	42.3 – 78.3
18	*Markhamia obtusifolia*	Stem bark	346.8	241.1 – 498.8
19	*Mystroxylon aethiopicum*	Stem bark	398.8	260.6 – 610.3
20	*Ochna mossambicensis*	Root bark	3.3	2.4 – 4.5
21	*Securidaca Longipedunculata*	Stem bark	55.3	41.8 – 73.0
22	*Senecio deltoideus*	Root bark	194.4	135.7 – 278.4
		Leaf	416.1	262.8 – 658.8
23	*Spirostachys africana *	Stem bark	4.4	3.0 – 6.5
24	*Trichodesma zeylanicum*	Leaf	2045.0	1440.0 – 2904.0
		Root bark	188.4	136.3 – 260.6
25	*Zanthoxylum chalybeum*	Root bark	38.5	32.5 – 45.6
		Stem bark	26.3	22.0 – 31.4
26	*Catharanthus roseus* (Positive Control)	Leaf	6.7	4.6 – 9.6

## Data Availability

Materials and data [Voucher specimens for plants] obtained in this study are available at the hebarium of Institute of Traditional Medicine, Department of Natural Products Development and Formulations, Muhimbili University of Health and Allied Sciences, P.O. Box 65001, Dar es Salaam, Tanzania. [Study report] Reports of this study are available at the Institute of Traditional Medicine, Department of Natural Products Development and Formulations, Muhimbili University of Health and Allied Sciences, P.O. Box 65001, Dar es Salaam, Tanzania. and at The Government Chemist Laboratoty Authority (GCLA) of Tanzania, P.O. Box 164 Dar es Salaam, Tanzania. upon request from the corresponding author.
